# Implementation of eMental Health care: viewpoints from key informants from organizations and agencies with eHealth mandates

**DOI:** 10.1186/s12911-017-0474-9

**Published:** 2017-06-02

**Authors:** Lori Wozney, Amanda S. Newton, Nicole D. Gehring, Kathryn Bennett, Anna Huguet, Lisa Hartling, Michele P. Dyson, Patrick McGrath

**Affiliations:** 1 Centre for Research in Family Health, IWK Health Centre, Halifax, NS Canada; 2 Department of Pediatrics, Faculty of Medicine & Dentistry, University of Alberta, Edmonton, AB Canada; 3 Department of Clinical Epidemiology and Biostatistics, and Offord Centre for Child Studies, McMaster University, Hamilton, ON Canada; 4 Department of Community Health and Epidemiology, Dalhousie University, Halifax, NS Canada; 5 IWK Health Centre, 5850—5980 University Avenue, Halifax, NS Canada; 6 Department of Pediatrics, University of Alberta, 3-526 Edmonton Clinic Health Academy, 11405-87 Avenue, Edmonton, AB Canada T6G 1C9

**Keywords:** ehealth, Mental health, Implementation science, Health planning, Telehealth, Organizational innovation, Behavior change wheel, Decision-making, Health organizations

## Abstract

**Background:**

The use of technology such as computers, tablets, and smartphones to improve access to and the delivery of mental health care (eMental Health care) is growing worldwide. However, despite the rapidly expanding evidence base demonstrating the efficacy of eMental Health care, its implementation in clinical practice and health care systems remains fragmented. To date, no peer-reviewed, key-informant studies have reported on the perspectives of decision-makers concerned with whether and how to implement eMental Health care.

**Methods:**

From September to November 2015, we conducted 31 interviews with key informants responsible for leadership, policy, research, and/ or information technology in organizations influential in the adoption of technology for eMental Health care. Deductive and inductive thematic analyses of transcripts were conducted using the Behavior Change Wheel as an organizing framework. Frequency and intensity effect sizes were calculated for emerging themes to further explore patterns within the data.

**Results:**

Key informant responses (*n* = 31) representing 6 developed countries and multiple organizations showed consensus on common factors impacting implementation: individual and organizational capacities (e.g., computer literacy skills [patients and providers], knowledge gaps about cyber security, limited knowledge of available services); motivational drivers of technology-based care (e.g., extending care, data analytics); and opportunities for health systems to advance eMental Health care implementation (e.g., intersectoral research, rapid testing cycles, sustainable funding). Frequency effect sizes showed strong associations between implementation and credibility, knowledge, workflow, patient empowerment, electronic medical record (EMR) integration, sustained funding and intersectoral networks. Intensity effect sizes showed the highest concentration of statements (>10% of all comments) related to funding, credibility, knowledge gaps, and patient empowerment.

**Conclusion:**

This study provides previously unavailable information about key informant perspectives on eMental Health care implementation. The themes that emerged, namely the need to intensify intersectoral research, measure/monitor readiness to implement, define cost-utility benchmarks, raise awareness about available technologies, and test assumptions that ‘proven’ technologies will be easily integrated can inform the design and evaluation of eMental Health care implementation models.

**Electronic supplementary material:**

The online version of this article (doi:10.1186/s12911-017-0474-9) contains supplementary material, which is available to authorized users.

## Background

Fifteen years ago Eysenbach posited that eHealth, in a broad sense “characterizes not only a technical development, but also a state-of-mind, a way of thinking, an attitude, and a commitment for networked, global thinking, to improve healthcare locally, regionally, and worldwide by using information and communication technology” [1]. Today, the term eHealth care is used to describe use of the Internet and related technologies such as computers, tablets and smartphones in the delivery of health care. The opportunities afforded by technology for mental health care, ‘eMental Health care’, to improve access to and the delivery of mental health care around the world are widely recognized [2] and the American Psychiatric Association (APA) has noted an intensifying commitment to using eMental Health technologies to deliver care [3].

Recent meta-analytic evidence from 63 countries suggests a global lifetime prevalence rate of approximately 29% for common mental disorders [4] and a high burden of disease and resulting disability. It is not surprising then, that eMental Health care technologies have been/are being developed for the treatment of many mental health conditions (e.g., anxiety, depression, bipolar disorder [5–9]). Individuals on their own may access such technologies so that they can receive psychological therapy (self-guided, technology-based therapy) or health care providers may access a technology so that they can deliver mental health care (partly self-guided, technology-assisted therapy). Though not universal, both short- and long-term positive outcomes have been reported for a diverse range of eMental Health technologies [10, 11]. There is a growing consensus, however, that health systems must evolve in order to exploit the potential of technologies to improve mental health care worldwide.

Individuals with mental health conditions are increasingly turning to the Internet for information, advice, support, and to share their experiences [12]. They are increasingly expect eMental Health options as part of their care [13, 14] and report high levels of technology device use [15] in their everyday lives. With three quarters of all lifetime cases of mental health conditions beginning by 24 years of age [16], and research suggesting youth view current/traditional mental health services negatively [17], there is interest among decision-makers in harnessing eMental Health opportunities for emerging generations especially. Yet, to catalyze the potential of mental health care reform, eMental Health care requires a new ‘way of thinking’ across multiple levels—patient, provider, administrative, and policy. This multi-level integration and commitment has the potential to improve patient experiences and health outcomes, create operational efficiencies in the delivery of care, and build provider capacity [18–20].

While the increasing evidence base supporting the efficacy and effectiveness of eMental Health care technologies from the patient perspective grows, the implementation of technologies into clinical practice remains slow and fragmented [21–24]. Ruwaard [25] has framed the situation adeptly saying “what was tested remains unimplemented and what is being implemented has not been tested” (p [26]). Effective and innovative eMental Health care integration requires sufficient motivational drivers [27] particularly from health care providers and policy makers. Previous research has shown that skills in collecting, appraising and disseminating research evidence are not enough for transforming research knowledge into clinical action. An understanding of professional behaviour, local context, personal and organizational development, change management and diffusion of innovation all impact health systems’ capacity to implement eMental Health care technologies [28, 29]. The remodeling of workflow and job design across interconnected mental health professionals and processes [30] is required to realize the potential of eMental Health care for population-level health benefits. Knowledge transfer among stakeholders would allow patient-level research evidence to be considered together with the perspectives, experiences and outcomes of all groups affected by future implementation decisions [31]. Barriers to this kind of organizational learning and change persist.

Key informants interviewed by Whittaker in 2010 on eHealth implementation (broadly, not specific to mental health) showed that policy makers, administrators and organizational leaders in the United States viewed eHealth care as potentially transformative in patient-centered care [32]. However, there were differences in informant views on the role of research trials, governance structures, and security solutions in advancing implementation efforts. Specifically, their study highlighted diverse opinions on the optimal research design methodologies to improve system uptake and the extent to which government agencies should be involved in using ehealth data for population health surveillance. In 2013, Jones and Ashurst conducted an educational online discussion forum with stakeholders self-selected from a website and focused solely on stakeholder concerns [33]. A major finding of their study was that patient and provider choice in methods of communication (online or not) was identified as an easy adjunctive way of starting to integrate eHealth for mental health care. We found no peer-reviewed key-informant or stakeholder studies since that have identified and mapped the perspectives (capabilities, motivations, and opportunities) of decision-makers in the implementation of eMental Health care specifically. Because technological innovations and how people use them is rapidly evolving, prospective studies of this topic area must be conducted and published regularly to keep up with the ever-changing landscape [34]. In this paper we build on previous work to ascertain not only what is currently being done internationally to implement eMental Health care, but also reasons for misunderstandings around key implementation issues. We conducted a qualitative study with key stakeholders to address three main objectives: 1) To identify current eMental Health care implementation processes and adoption strategies by public sectors; 2) To explore descriptions of organizational and individual readiness to adopt eMental Health technologies; and 3) To evaluate the frequency and intensity effects of reported implementation factors.

## Methods

This study involved in-depth individual interviews with a multi-national group of key informants using purposive and snowball sampling strategies. Interviews were conducted between September 2015 and November 2015.

### Sample

We conducted a three pronged search to identify potential interview participants: 1) targeted Google searches for relevant government, health, and technology organizations in developed countries with the largest eHealth markets (Canada, Australia, the Netherlands, New Zealand, the United States, and the United Kingdom) [35], 2) rapid review of recent literature in pertinent topics, and 3) recommendations from members of our research team. Participants needed to be fluent in English. Relevant organizations and government affiliated agencies were chosen based on having clearly stated goals and/or funding relating to eHealth and behavioural technologies, and in the case of academic bodies, relevant published research associated with these topics. These bodies included the Mental Health Commission of Canada’s e-mental health steering committee, the Australian Government’s Department of Health and Ageing e-mental health expert advisory committee and National eHealth Transition Authority, New Zealand’s National Health IT Board, and the United Kingdom’s MindTech Healthcare Technology Co-operative. We identified individuals responsible for leadership, policy, research and information technology that are influential in the use of eMental Health technologies. Many individuals were specifically singled out due to their role as an eMental Health or eHealth advisor on a number of policy related reports and documents.

Overall, we created a list of 91 potential informants. From the organizations and institutions chosen, individuals who were actively developing or have developed eHealth technologies, were high ranking in their particular field (e.g., Professor, Director, Chief Executive Officer, etc.), and who focused on eMental Health were prioritized. These individuals were considered to be knowledgeable and experienced in their roles within their specific domain and able to provide intimate knowledge and experience on the subject area. To maintain equitable representation, potential key informants were systematically categorized as academic, government, organization/association, and industry representatives and were allocated based on their country of practice. Four potential informants were chosen from each country and representing each category before approaching additional potential informants. In addition, at the end of each interview, participants were asked if they could recommend additional key informants that we should consider interviewing. This was an iterative process until the number of key informants who participated in this qualitative study was the recommended mean number of 30 participants (recommended range: 12 to 60 participants) [36, 37]. Potential participants were contacted directly by e-mail or via e-mail introductions from others.

### Interviews

Based upon a literature review and discussions with the research team, a semi-structured interview guide (see Additional file 1) was developed in the absence of a guiding theoretical framework to allow more flexibility in the examination of opinions, attitudes, and perspectives on current eMental Health implementation approaches. The guide was reviewed by the research team for face and content validity as well as feasibility (e.g., time to complete). The guide was then pilot tested by one investigator (NDG) with a convenience sample of 2 experts and 2 non-experts external to the research team. The investigator conducted practice interviews with these individuals to gain ease with asking the interview questions, flow and timing, and feedback was requested on the questions asked. The first interview provided the opportunity to refine, rephrase and clarify some questions in the guide. While in some questions, prompts were added in order to acquire better answers. The pilot test also helped to draw attention to questions that ought to have been included in the guide, for example, a question pertaining directly to governmental policies was added. Subsequent pilot interviews (*n* = 3) validated the changes made as evidenced by the nature of answers and information obtained from the remaining interviewees. Attention was also paid to the time required to conduct each interview. The final interview guide was designed to facilitate interviews lasting 30–60 minutes and covering four domains: 1) informant demographics, 2) current eMental Health technologies, 3) implementation strategies and, 4) adoption readiness. During the study, the interview guide was modified and refined on the basis of previous interviews to further explore emerging key issues in subsequent interviews.

All interviews were conducted by one of the investigators (NDG). Key informants were free to deviate from the guide and the interviewer intervened only to clarify issues or introduce a new domain. The guide was e-mailed to key informants two days prior to their scheduled interview to allow time to review the questions. Interviews were conducted over the phone or via Skype, depending on the informant’s preference and were digitally recorded with consent and later transcribed verbatim by an experienced transcriptionist. All informants provided verbal informed consent prior to the interview and the study protocol was approved by the University of Alberta Research Ethics Board.

### Data analysis

Data analysis proceeded in three phases:

- ***Phase 1 (Thematic Analysis)*** In accordance with Strauss and Corbin (1990) [38], a line-by-line analysis of the transcripts was conducted by one of the investigators (NDG) in order to generate an initial list of potential themes. Results of this initial reading were discussed with our interdisciplinary team to refine coding procedures.
- ***Phase 2 (Content Analysis)*** An integrative approach was used to code interview data that included both deductive and inductive methods [39]. An initial deductive analysis involved the identification and grouping of similar data together into three *a priori* themes derived from the Behaviour Change Wheel (BCW) [40]. The BCW has the benefit of having been derived from 19 other models and classifications already available and therefore covering concepts that have previously been considered to be important to implementation. Through this model our research question could be conceptualized as: ‘What conditions internal to health systems and in their social and physical environment need to be in place for eMental Health implementation targets to be achieved?’. Specifically, the BCW provided a lens through which to consider the data in terms of the 3 essential conditions for successful behavior change systems: ***capabilities*** (i.e., psychological and physical capacity to implement eMental Health services, including the necessary knowledge and skills), ***motivations*** (i.e., reflective and automatic mechanisms that activate or inhibit implementation), and ***opportunities*** (i.e., physical and social environment that enables implementation into health care systems). Inductive analysis followed using open coding to categorize data into subthemes. Constant comparisons of the coded sections of the transcripts with each other and with the emerging themes allowed further refinement. All coding was done by one of the investigators (NDG). In addition, several joint coding and analysis sessions involving other investigators (LW, ASN) were conducted to increase theoretical sensitivity and to ensure high quality of coding.
- ***Phase 3 (Intensity and Frequency Analysis)*** An analysis of the relationship between reported themes was conducted by calculating of frequency and intensity effect sizes as suggested by Onwuegbuzie [41] and Sandelowski [42]. This allowed an aggregation approach to accommodate the distinctive features of informants’ viewpoints. Seeing all of the findings pertaining to one theme together in this way preserves the complexity of the findings and optimizes the descriptive validity of the thematic analysis [43]. Furthermore, because research suggests that concerns which consume attention often have a disproportionately large impact on the judgment process [44], it is reasonable to conclude that the frequency and intensity in which topics are identified is useful information for decision-makers. A *frequency effect size,* a measure of how dispersed the themes were across interviewees, was computed by taking the number of interviews coded for the theme and dividing this number by the total number of interviews. An *intensity effect size*, a measure of theme concentration, was derived by dividing the total number of references to each theme by the total number of references across all interviews. Percentile ranks (pR) were calculated for all effect size to better describe characteristics of the distribution of themes. Strong association of a theme was classified as a pR > 75%; Moderate as pR >25% ≤ 75%; or weak as pR ≤ 25%. This classification was selected on the basis of conventional normed assessment categories and followed the model provided by Wao, Dedrick & Ferron [45].

A summary report of key findings (organized into themes and subthemes) was provided to key informants for comment, and some amendments were made based on their feedback. Thematic and content analyses were conducted using NVivo software (QSR International). Effect sizes and percentile ranks were computed using SPSS software (IBM).

## Results

Of the 73 individuals we contacted, 19 did not respond to our request for study participation (20.8%) and 16 individuals declined study participation (17.6%). Of the 38 individuals that agreed to participate, 7 did not complete an interview due to scheduling conflicts. In total, 31 key informants participated in our semi-structured interview (resulting in a 42% response rate). Key informants were from Australia (*n* = 5), Canada (*n* = 5), the Netherlands (*n* = 4), New Zealand (*n* = 7), the United Kingdom (*n* = 5), and the United States (*n* = 5). Collectively, the average years working in the eHealth field among the informants was 11.42 years (*SD*: 9.38, Range 2–48). Key informants were academics working in eMental Health research (*n* = 13), from organizations that sponsor eMental Health initiatives or research (*n* = 9), representatives from national government agencies with an interest in eMental Health care (*n* = 6), and representatives from eMental Health companies (*n* = 3). The majority of key informants (*n* = 19) provided written consent to be listed in the publication of study results (see Additional file 2). The average length of the interviews was 39 minutes (*SD* = 10.06, Range 22:32–57:51).

### Developing capacity for eMental Health knowledge mobilization

Key informants identified 12 unique physical and psychological capacity gaps within health care systems that hinder mobilization of eMental Health initiatives (see Table 1).

**Table 1:** Description and examples of themes

Theme	Description of the Theme (D) and Example of Statements (E)
Capacity	
Broadband	D: Heterogeneity in broadband access E: “So broadband speed and availability of broadband around the country, it varies, so a lot of people say get it online, get it online. I think that works if you’re in cities, with good technology and there are some…where getting access is really difficult.”
Change	D: Speed at which technology is evolving E: “With [technology] moving fast, the strategies might become outdated but also if it’s not part of a big change program, then I guess the strategy could be ignored and there could still be surprise pop-ups or emerging things that take it in a different direction.”
Credibility	D: Uncertain credibility about how technology works E: “So in that sense, yes we might say that a certain treatment, based on CBT [cognitive behavioural therapy] for what I know of is effective, but we actually do not really know why and so that’s a bit of the scary part.”
Cyber Security	D: Privacy protection issues around personal health information E: “There needs to be some really clear guidelines or mandates, legislation that states exactly, if there are going to be mental health conversations occurring, who is able to look at that outside of the practitioner and the client?”
Engagement	D: Patient engagement with eHealth is low E: “Interestingly I think the barriers are not technological or even resource based. I think the barrier is actually a much more ancient problem and that’s getting people to care enough about their health to do something.”
Insecurity	D: Insecurities about work obsolescence and employment E: “I think that there’s potentially a bit of professional insecurity there as well. You know, with the availability of these programs, it’s kind of saying, you invested all these years in your own professional training and now there’s a program that can do the job that you did without any need for you anymore.”
Interference	D: How technology changes and ‘interferes’ with patient relationship E: “…not wanting the technology to kind of take the human element out of clinical treatment and so you get a lot of different kind of attitudinal and kind of negative reactions and a lack of openness in some cases.”
Knowledge Gap	D: Lack of knowledge about existence and effectiveness of available eHealth technologies E: “I think the biggest barrier is just a lack of knowledge that these things actually exist and that in many cases they can be as effective as face-to-face treatment and you know. I think it’s just a matter of there being a bit of a gap of knowledge about these things existing.”
Literacy	D: Levels of computer literacy for patients and providers E: “Some providers and some decision makers are early adopters and others are, you know, very much just kind of comfortable with their practices and don’t really see value or see that an introduction of technology is more troubling than helpful and for a number of different reasons, you know, it can be just not wanting or not having the capacity to learn a new system.”
Marketing	D: How eHealth technologies are marketed and promoted E: “We need to be able to tell the story of those successes so that more and more people become aware of the potential of this resource.”
Product	D:Treating eHealth as products as opposed to services E: “I think that organizations are really going to have to take this on and then I think that people who are interested in the development of eHealth are going to have to think about how they…how they partner with those organizations to be able to build eHealth interventions that are kind of tailored to the different problems that those organizations encounter.”
Workflow	D: How technologies change provider workflow E: “If the technology adds more work (paperwork, bureaucracy, etc.) to the employee that’s it. I mean if it’s not part of the clinical workflow, it doesn’t matter what is it, it’s never going to work”
Motivation	
Big Data	D: Ability to use data analytics to inform practice (at patient or public health level) E: “I think improving the records keeping and sharing of data, I think has the potential to change things quite dramatically but in terms of allowing the health service…to look after people more effectively because they’re able to get the picture about what’s going on, but then there’s a big trend…at the moment for the idea of giving patients access to their own information, which is sort of happening, albeit quite slowly, and I think we really see this within our work as something that service users in mental health really want.”
Blended Care	D: Role of eMental Health in stepped and blended care E: “Increasing access way beyond the capacity of parent services requires us to looks at blended care models and that’s really where online technology can increase capacity of our health systems.”
Cost/Benefit	D: Economic benefits of eMental Health and costs of delivery E: “The potential for spending on health way outweighs the number of dollars available, so I think it’s thinking about how we can use it in a smart way. I think the dilemma is finding a balance between finding a place for it, but not shortcutting things so that we don’t just say, oh we don’t need therapists anymore because it’s cheaper”
Empowerment	D: Empowering patients to engage in managing their own care E: “We need to break down the barriers to sharing information when the client wants it shared and have it not mandated by government.”
Electronic Medical Record	D: Role of electronic health records in eMental Health care E: “Implementation of meaningful use around electronic health record…I think that’s definitely one facilitating eMental Health strategy and you know, we’re starting to see mental health and behavioural health indicators added into EHR [electronic health record] systems more and more, so that’s one piece of it.”
Unreached	D: Providing access to eMental Health care for people who might otherwise not have it E: “There’s way more people who could benefit from assistance than we have the resources to help in person and a lot of people, and the research bears this out…it’s having that access to a professional online that makes the difference.”
Wait Times	D: Improving health system inefficiencies with shorter wait times E: “Because the reality is people are waiting really long times to get care right now and so we have to think about a kind of system transformation that includes technology”
Opportunity	
Alignment	D: Align programs and initiatives with policy objectives E: “The only way bureaucrats like to fund programs, initiatives, if it meets their policy objectives and a lot of times when I am sitting in an environment where I'm simply leading innovation and driving innovation, I don’t think of policy right off the bat, because it’s not transparent”
Endorsement	D: Develop guidelines to support superior projects E: “Some [eHealth technologies] are really superior and some aren’t very good, let’s be frank about it. And it’s really trying…what would be helpful is teasing out and supporting the ones that are very good.”
Funding	D: Need for sustainable funding E: “I mean that’s the only way I can think of it being really robust is that, you know, there’s policy statements that support it as an integral part of the health care system and then there’s funding directed to it because if there’s not funding directed to it, it just becomes a bit on the fringe.”
Incentives	D: Lack of incentives for adoption and use E: “Give professionals more time to get used to a program. So you could reward their attention to adopt a new way of working by giving them more time off or giving them more incentives to get used to the new method.”
Infrastructure	D: Technology tools (software, hardware) that allow eMental Health delivery E: “The approach we’re taking is that if we’re building stuff, we’re building it on open source so you don’t have that lock in and you can reuse and repurpose it. So we’re trying to build a sustainable approach, which means you don’t have to get locked into a provider each time.”
Licensing	D: Absence of national licensing system (United States specific) E: “For instance, I know that you can’t treat patients across states, so because of different legislations that may apply. So your license doesn’t apply to the other state, even though you could treat patients in other states over the Internet.”
Mandate	D: Guidelines, mandates and legislation E: “There needs to be additional policy, additional clarification around the regulatory aspects of…and confidentiality aspects of using eHealth tools, either within or outside of the clinical setting.”
Networks	D: Build stronger networks between academics, professionals, health providers and end users E: “I feel there just seems to be such a gap between what’s happening in the academic world and what’s happening at the community, kind of delivery level and also what people are saying they need. We need all components involved: academics, end users, health providers, etc.”
Partnership	D: Encourage public-private partnerships E: “Working with the private sector because they’re way ahead of us in lots of ways and in other ways they’re not because [academics] have the content knowledge, we have the experts but they have the resources and the technology.”
Patient Cost	D: Coverage of eHealth services E: “The government and the insurance companies need to recognize that the proven technologies should be paid for. They should be part of a coverage that a person has on their insurance program.”
Reimbursement	D: Unclear reimbursement model for patients and providers E: “If you can’t get it reimbursed, if people have to pay for these kinds of treatments themselves, they won’t come at all because if you can get something for free, face-to-face, why should you pay for it online?”

Ensuring that new technologies are streamlined within existing health system workflows was regarded as essential to increasing uptake. However, informants suggested the assimilation of eMental Health technology requires extensive ***change*** to systems, structures, and individual ***workflow*** as innovations have both technical (e.g., hardware, bandwidth) and peripheral (e.g., human resources, job design) implications. This challenge was identified as affecting both the way providers are used to working and the way patients are used to engaging with the health system. The danger perceived by informants was that eMental Health technologies were still seen as isolated from the wider provision of services meaning routinization and full integration of a technology by providers is still only being partially achieved.

Key informants discussed how technology can disrupt or ***interfere*** with traditional patient-provider relationships and workflow in a manner that may produce an impersonal and inferior patient-practitioner dynamic. As one informant stated, “you know, not wanting the technology to kind of take the human element out of clinical treatment and so you get a lot of different kind of attitudinal and kind of negative reactions and a lack of openness.” The difficulty in forecasting the peripheral implications for each provider and ensuring eMental Health technologies become part of the routine workflow was seen by many informants as a key challenge. As one informant noted:

“deploying a system is easy but making workforce development and creating incentive programs and creating awareness amongst the clinical community and particularly primary care, it doesn’t matter what country it is, is the tricky part. [Professionals] are enshrined in a particular way they work… Any change of workflow is a challenge…if whatever we do does not fit within the clinical workflow of the practice in the real world and what [general practitioners] are doing, they’re not going to use it.”

Sensitivity to the local patient and provider “ways of working” was seen as essential, but the informants generally agreed that full assessment and ongoing tailoring of eMental Health technologies within clinical practice did not always occur in practice. Hybrid implementation (eHealth as adjunct to face-to-face) was discussed as a potentially less disruptive approach as it resembles current workflow providing an easier learning curve for providers and patients. While heterogeneity within ***broadband*** (i.e., areas of low connectivity, cellular carriers) and mobile technologies (e.g., iPhone versus Android) were identified by some as being a potential challenge to hybrid implementation, this was not a widely expressed concern.

Even when providers and patients are “ready” to change workflow and clinical practices in order to use eMental Health services, informants described a lack of ***knowledge*** of the current eMental Health care landscape, including what services and technologies are available, ***credible*** and safe. Most informants identified lack of awareness broadly as a significant barrier to the adoption of technologies to serve mental health conditions. Alternately, informants reported relatively low public awareness of effective eMental Health services and poor ***marketing*** of available technologies, which in turn reduces demand. They noted that prevailing social norms and messaging continue to situate eHealth as a “***product***” rather than a legitimate health care service and advocated for improved public marketing and eMental Health education. From this perspective, key informants felt that as patients become increasingly aware of eMental Health services, and empowered to choose eHealth options, public ***engagement*** will dictate policy reform.

***Computer literacy*** skills and related emotional reactions to ***job insecurity*** were identified by key informants as rate-limiting areas of eMental Health implementation. Professionals resistant to change, uncertain of the value of technology, and stringent on established processes can generate a digital divide between early adopters and laggards.

For example, one informant stated,

“there’s a huge amount of computer literacy, we’ve come a long way, but then there’s pockets of non-literacy for both professionals and patients alike, how do we deal with those gaps? … there are some barriers in terms of the way that people feel like accepting technology as well, and feeling like it’s…imposed on them.”

while another mentioned,

“the age of the health workforce and varying computer literacy… I don’t think peoples’ jobs are on the line but the perception is that they are and…there’s definitely an element of professional pride being threatened and it’s a hard pill to swallow for some.”

In this regard, some key informants described the importance of integrating eHealth training early in educational pursuits and career development for all health practitioners, while several other informants described that as the global age raises, these technological shortcomings may dissipate.

Key informants identified a knowledge and expertise gap around ***cyber security*** as a noteworthy obstacle to large-scale implementation. These included insufficient privacy protection around personal health information and how this may be viewed and defined differently across governing bodies (e.g., professional accreditation organization, state/provincial governments, universities, industry, countries, etc.)

“government institutions or health authorities find it very hard to get their heads around privacy and risk issues related to eHealth, but then you have the private companies and entrepreneurs of the world wanting to go full steam ahead with less bureaucratic roadblocks in the way, and then you see these two worlds meeting, but the health care system or government for that matter can’t catch up around some of the regulatory and policy issues because they have more at risk when it comes to breaches in security and privacy.”

With key informants looking to governing bodies for guidance, some informants stressed the need for established protocols, specifically in terms of health data owner- and/or stewardship (i.e., patient, government, or organizational level).

### Motivations to transform the delivery and quality of mental health care

Although key informants focused on barriers and challenges with implementation and adoption, seven motivating factors were identified as ways to energize and catalyze actions towards implementing eMental Health care (See Table 1).

Key informants asserted that health systems are placing a growing priority on eMental Health care as it can increase quality of care in ***blended care*** models where technology is used in addition to face-to-face care. In this capacity, eMental Health was seen as ultimately reducing ***wait times***, reaching people who would otherwise be ***unreached*** and increasing capacity of health care systems. As one informant discussed, “all different stakeholders are aware that, and emphasize that they think blended care has more potential than, for instance, only online self-help and so eMental Health really has the capacity to scale up routine practice.” There was some concern, however, that:

“we need to be careful not to get distracted with technology because technology is the tool rather than the therapeutic mechanism and I think we, as a field, we can get distracted with hype, and that might be fine for funding agencies who want to hear about the latest innovation using the latest widgets. But that then probably means we sometimes under deliver therapeutic benefits for patients.”

Integration with ***electronic health records*** was identified as one way to increase delegation and coordination of care. As one informant stated:

“potentially very useful is called patient controlled electronic health record…it could be the point of access to a lot of measurable eHealth tools [and] if it was connected then to an [aggregated mental health patient portal] and could have algorithms to actually point them in the right direction as far as the tools…so that they can actually access information and help themselves.”

Additionally, eMental Health care was perceived as addressing restraints from funding agencies by providing a substantial ***cost-benefit***: “I think [eMental Health] can clearly reach large numbers of people quickly and cost effectively in a way that face-to-face psychiatry or psychological interventions can’t.” However, while eMental Health can be cost-effective for health systems many key informants echoed the position that, “developing these interventions is quite expensive and then trying to keep them up-to-date is quite expensive and keeping them running seamlessly day in, day out is quite challenging.”

Finally, key informants discussed eMental Health as a means of increased accessibility of “***big data***”: “[eHealth] already has a large benefit in the collection of big data. That’s without a doubt. More importantly, is what we can learn from the information that’s collected and using that data to further increase patient ***empowerment*** in managing their own care.” One informant also believed that the sharing and utilizing data, particularly in terms of implementation data (i.e., reach, adherence, impact), across a range of different programs would catalyze more efficient implementation across systems. Furthermore, two informants from the United States discussed how the proliferation of technological innovations, such as the ‘always-on’ nature of mobile devices and wearable technology, and geolocation technology can allow the rapid analysis of data to be used to relay passive contextual, geographic and behavioural information:

“I’ve come across different innovations in keeping up with this industry and there are a couple of things that we’re not doing but others are. One is to try and find ways to do tracking of a person’s mood or mental health status or happiness or you know…in a way that isn’t requiring of daily input from a user. So…trying to figure out ways of having a device like a Smartphone, being able to track your movements…be correlated with certain things like certain mood states and what not.”

### Opportunities for furthering eMental Health implementation

Key informants described how opportunities for implementation were limited or advanced by physical (e.g., funding, digital infrastructure, billing/prescribing) and social (e.g., changing ideas about ‘evidence’, length of research cycles, policy mandate) context factors. Eleven opportunity related themes were identified (see Table 1).

Key informants posited that the lack of digital ***infrastructure*** was an ongoing implementation-limiting factor. They emphasized that delivering an eHealth service was an ongoing cost, not a one-off. Effective, integrated work between health care and industry to build these sustainable systems was often perceived as uncoordinated. Successful access to eMental Health models was regarded as contingent upon these issues and the need to address interoperability not just at the provider level (e.g., electronic medical records (EMRs), communicating with third party patient portals, data formats), but also at the patient level. Key informants conceded unclear ***reimbursement*** and ***licensing*** models for eMental Health care services as another key feature. At a basic level making sure users can complete transactions or processes online was described as necessary. Although levels of universal health care and health insurance models differed across the countries represented by informants, there was generally reported spotty and vague coverage of eMental Health care, which was viewed by informants as a barrier for patients:

“you know, if there is an [eMental Health] service out there, but a patient can’t get reimbursed for using it and has to pay out of pocket, that patient may ultimately feel that that service might be preying on them. If the government or insurance company doesn’t financially support the service, then a patient may think they are just out for profits, while a free product doesn’t hold much value either because who’s regulating quality?”

Findings suggested that unreliable coverage initiates barriers for physicians to refer patients to eMental Health technologies because they do not want the ***cost to patients*** to come from out-of-pocket. One key informant, a general practitioner from Australia, highlighted the critical importance of billable eHealth “prescriptions” in streamlining this process, a model already established in Australia:

“allowing a [general practitioner] to write an [eMental Health] prescription where a patient can then go home log into their patient portal and type in the prescription code… now the [general practitioner] can really see if the patient has logged in, done an assessment, see what the score is and those kind of things, but now they can actually bill for that prescription.”

A related opportunity to advance implementation was concerned with ***incentivizing*** uptake: “So you could give professionals more time to get used to a program. So you could reward their intention to adopt a new way of working by giving them more time off or giving them more incentives to get used to the new method.”

The occasional overstating, lack of evidence or uncertain credibility about therapeutic effect came into question by all key informants, for example: “there’s tons out there and I think in most cases we don’t know [the credibility] because they haven’t been adequately tested.” The potential benefits of new technologies should be made transparent through ongoing evaluation and feedback so that new technologies have credibility and ***endorsement*** and are not just seen as novelty items.

There was also considerable discussion about the develop-test-implement cycles of new technologies and persistent difficulties in getting evidence-based treatments scaled up so multiple providers can make use of them:

“I think with the current model we are essentially using the traditional scientific model where one team develops something and then they spend a couple of years testing and another year or more writing it up and then they spend a year or more getting it online than then either you don’t get any implementation data at least for another year or two after that. One, that’s way too slow and two, it’s not an appropriate model in this sort of fast changing technology world.”

Key informants envisioned possible improvements to this testing/implementation cycle by building stronger ***networks*** between academics, professional health providers, end users, and encouraging public-private ***partnership*** **s**:

“There seems to be such a gap between what’s happening in the academic world and what’s happening in the community, kind of delivery level and also what people are saying they need. If we involved all stakeholders [academics, health providers, end users, private sector] right from the beginning we could probably avoid making a product that we just hope is relevant for someone; I think we forget to add that bit.”

Informants also proposed that ***funding*** for eMental Health services could be addressed through mandated cost-benefit analyses of new technologies, and government subsidies of the best available eMental Health practices. Moreover, several informants discussed hesitations in funding allocation, which lack attention to sustainability models. Without such, developed projects can be abandoned given lack of resources for system updates, hosting and marketing costs, and product upgrades which ultimately determine the relevance of innovations due to rapidly changing improvements and demands in today’s technological landscape.

Informants further proposed that policy ***mandates*** to support eMental Health care could be achieved through strategy development or execution of preliminary conceived strategies. Many key informants were aware of national eMental Health strategies shaping the implementation of eHealth. As one informant summarized:

“Our then government decided to develop this strategy and there were three planks in the strategy. The first was to develop what they called an eMental Health portal, which was a national resource, basically a web page that anyone in Australia could access…to find out what eMental Health products and resources were freely available The second plank in this commonwealth government’s strategy was what they were calling a virtual clinic, which basically would provide free online treatment for anyone in Australia…so the high prevalence disorders…and then the third plank in that strategy was a piece around implementation and integrating eMental Health into primary care.”

However, they went on to say that this policy is still an area of weakness at the moment due to changes in governing bodies: “There’s a massive need for reform. There’s a lot of inefficiency in the system, a lot of duplication and a lot of insecurity and uncertainty at the moment.” Similarly, some key informants were aware of a framework, however it did not manifest into national policy reform: “certainly we have an eMental Health framework …some years ago, which is still unpublished. So that’s kind of been stopped with the bureaucracy for a little while and some of that, as well, they restructured…so we haven’t got an overarching strategy in that sense.”

There was some skepticism as to whether the necessary political commitment on the part of both national and international decision makers is substantial enough to actually implement change. A similar pattern was described by other key informants who were aware of the initial developments of a strategy:

“I know the Mental Health Commission was working on one but I don’t know exactly what happened to it and I think they cancelled the meetings where they were kind of coming up with some of that. They have done some reporting on eMental Health and then I know that there’s, I’ve seen some strategies for specific groups but I don’t know that there’s a strategy.”

Further, policy reform recommended by key informants included clarification of practice guidelines, mandates, and legislation surrounding privacy policies, as well as an ***alignment*** of eMental Health programs and initiatives with clear-cut objectives.

### Frequency and intensity analysis

Table 2 presents the frequency effect sizes (FES) and the corresponding percentile rank (pR) with eMental Health implementation for each of the emergent themes. Based on the magnitude of the FES across all informants (*n* = 31) the *capability* themes of credibility, knowledge, and workflow were perceived to have strong associations with implementation. Within the *motivational* themes patient empowerment and EMR integration had the strongest association. Funding and intersectoral networks were the only two *opportunity* themes that showed strong association with implementation. Combined FES showed that 9 themes were perceived to have minimal association with eMental Health implementation: 6 from the capability themes (i.e., broadband availability, patient engagement, interference, literacy, marketing) and 3 from opportunity themes (i.e., product, endorsement, licensing and patient cost). Of note, all 6 motivational themes had moderate or strong association. When compared to academics, government officials and industry representatives, organizational representatives’ (*n* = 9) responses showed a strong association between cyber security and implementation with a FES of 67% (compared to 31%, 17%, 33% respectively). Although only a small number of industry representatives were interviewed (*n* = 3), informants in that group perceived job “insecurity” to have strong association (FES = 67%) with eMental Health implementation. However, job insecurity was ranked as only minimally associated by organizational representatives (FES = 11%) and only moderately associated by government (FES = 17%) and academic (FES = 31%) groups.

**Table 2:** Frequency Effect Sizes of emergent themes by informant group

	Combined (*n* = 31)		Academic (*n* = 13)		Government (*n* = 6)		Industry (*n* = 3)		Organization (*n* = 9)	
Theme	FES	pR	FES	pR	FES	pR	FES	pR	FES	pR
Capability										
Broadband	16%	25	15%	30	0%	13	0%	23	33%	52
Change	39%	70	38%	73	33%	58	0%	23	56%	75
Credibility	71%	100	69%	100	67%	95	67%	80	78%	95
Cyber	39%	70	31%	60	17%	38	33%	55	67%	88
Engagement	6%	5	8%	12	0%	13	0%	23	11%	18
Insecurity	26%	45	31%	60	17%	38	67%	80	11%	18
Interference	16%	25	23%	47	0%	13	0%	23	22%	33
Knowledge	58%	90	54%	87	50%	77	100%	98	56%	75
Literacy	16%	25	8%	12	0%	13	33%	55	56%	75
Marketing	6%	5	8%	12	17%	38	0%	23	0%	7
Product	10%	10	23%	47	0%	13	0%	23	0%	7
Workflow	42%	82	31%	60	67%	95	67%	80	33%	52
Motivation										
Big Data	23%	40	8%	12	17%	38	0%	23	56%	75
Blended Care	39%	70	62%	93	33%	58	0%	23	22%	33
Cost/Benefit	32%	52	38%	73	17%	38	33%	55	33%	52
Empowerment	58%	90	46%	83	50%	77	100%	98	67%	88
EMR	42%	82	38%	73	50%	77	67%	80	33%	52
Unreached	35%	58	38%	73	33%	58	0%	23	44%	63
Wait Times	26%	45	23%	47	33%	58	0%	23	33%	52
Opportunity										
Alignment	32%	52	23%	47	67%	95	67%	80	11%	18
Endorsement	13%	15	8%	12	50%	77	0%	23	0%	7
Funding	68%	97	62%	93	50%	77	67%	80	89%	100
Incentives	19%	35	15%	30	17%	38	33%	55	22%	33
Infrastructure	35%	58	15%	30	50%	77	33%	55	56%	75
Licensing	16%	25	23%	47	0%	13	0%	23	22%	33
Mandate	19%	35	8%	12	17%	38	33%	55	33%	52
Networks	58%	90	38%	73	67%	95	67%	80	78%	95
Partnership	39%	70	15%	30	50%	77	67%	80	56%	75
Patient Cost	13%	15	15%	30	0%	13	0%	23	22%	33
Reimburse	39%	70	62%	93	17%	38	67%	80	11%	18

Percentages were rounded up to whole numbers

*EMR* Electronic Medical Record, *FES* Frequency Effect Size (expressed as %), *pR* Percentile Rank (expressed as rank out of 100^th^ percentile)

Table 3 presents the intensity effect sizes (IES) and the corresponding percentile rank (pR) and perceived strength of association with eMental Health implementation (Assoc) of each of the emergent themes. Based on the magnitude of the IES across all coded statements (*n* = 310) the highest concentration of statements related to funding (13%), credibility (11%), knowledge (10%) and patient empowerment (8%). In addition, change (6%), blended care (6%), networks (6%) and reimbursement (6%) also showed relatively strong associated concentrations. The lowest concentration of statements within a theme was concern over low patient engagement (1%) and the need to better market available technologies (1%).

**Table 3:** Intensity Effect Sizes of emergent themes from informant interviews

	Combined (*n* = 310)	
Theme	IES	pR
Capability		
Broadband	2%	25
Change	6%	80
Credibility	11%	97
Cyber	5%	72
Engagement	1%	7
Insecurity	3%	33
Interference	2%	20
Knowledge	10%	93
Literacy	2%	30
Marketing	1%	3
Product	1%	13
Workflow	4%	55
Motivation		
Big Data	3%	42
Blended Care	6%	80
Cost/Benefit	3%	50
Empowerment	8%	90
EMR	5%	72
Unreached	5%	62
Wait Times	3%	42
Opportunity		
Alignment	5%	67
Endorsement	1%	13
Funding	13%	100
Incentives	3%	42
Infrastructure	5%	62
Licensing	2%	25
Mandate	3%	42
Networks	6%	80
Partnership	4%	55
Patient Cost	1%	13
Reimburse	6%	87

Percentages were rounded up to whole numbers

*EMR* Electronic Medical Record, *IES* Intensity Effect Size (expressed as %), *pR* Percentile Rank (expressed as rank out of 100^th^ percentile)

## Discussion

Results from this study show sustainable eMental Health care requires action from multiple sectors in society, including the public at large. However, this study also signifies that among key informants at least, there is consensus on most of the priority issues and that they have a willingness to tackle them. Uptake and use of eMental Health has fallen far short of levels predicted by policymakers; perhaps, as our informant interviews revealed, this is because technology innovation has proceeded faster than committed exploration of the personal, clinical, organizational, and motivational implications [26]. Because of the sampling method and relatively small sample, it would not be appropriate to make generalizations about the results; however, the study provides up-to-date and valuable information for decision-makers and researchers in this field. There are four key take away messages for decision-makers from our study:

### Reimbursement and cost-tracking

Informants felt that there continues to be a lack of coherent, comprehensive national (and international) planning and strategic processes around how to determine if a given eMental Health service provides value for cost (at individual and population levels). Costing, billing and prescribing capacity was felt to be insufficient in all countries interviewed, suggesting significant work in that area remains. Provider billing and insurance coverage for patients was a problem in most of the countries surveyed. Deconstructing the financial complexity into manageable components and managing at that level is the necessary first step. Informants had a strong sense that sustained funding from governments and evidence of cost-utility analysis from researchers were still badly needed in the field of eMental Health to establish credibility. This position was echoed in the recent WHO 2016 report [46]. The ability to differentiate between development costs, research costs and ongoing service delivery costs is therefore a critical area of future research that needs to be informed by policy makers and administrators to ensure congruence between research measures collected and metrics required to justify deployment and insurance costs.

### The role of evidence in the demand-supply chain

There were conflicting impressions on the current pace of implementation and whether it was moving in the right direction. On one hand there was concern that research cycles take too long, that the traditional models of testing are outdated or lack authenticity in the ‘real world’ health care setting. Given the surge of commercial eMental Health care products being downloaded and purchased there is a concern that demand is exceeding evidence-based supply and if patients are already using these technologies then health systems need to “get on board”. On the other hand, there was concern about the safety, tolerability and efficacy of eMental Health technologies not being extensively understood and thus, might potentially put patients or providers at risk. Informants indicated that many providers are interested in incorporating eMental Health (i.e., mood apps, medication trackers, online courses, telepsychiatry) into their practice, but they perceive a lack of strong evidence base, accreditation or licensing structure that gives a governing framework in which they can provide these services to patients with reasonable assurance of effectiveness and safety [47]. While the technologies we need to deliver eMental Health are available to a large extent, including interoperability of information and the systems that share it, we do not yet have the flexibility of approach to collaborate and share “what works” or consensus around what kinds of evidence providers, policy-makers and patients prefer and require in order to make decisions about using eMental Health technologies.

### Assess eHealth literacy skills of all stakeholder groups

The belief that provision of eMental Health will automatically result in reduced costs was challenged by many informants as an example of misguided visions of “user friendly” technology that would require little training or support. Concerns with the level of societal and organizational readiness (e.g., computer literacy, technology acceptance, professional pride) were prominent among informant comments. There was a clear sense that despite the potential of eHealth it is still in competition with other priorities. In common with other complex service level innovations, the real challenge lies in implementation and routinization of the eMental Health services beyond initial adoption [48]. eMental Health services that only address readiness at the patient level (i.e., Do these technologies work and are patients satisfied with them?), and do not take into account the social and environmental influences (i.e., Are providers skilled enough to deliver care in this way?, How will these technologies change staff workflow?) are unlikely to work [40]. It appears mental health care organizations commonly find themselves caught between the organizational pressures for delivering eHealth and lack of capacity or even resistance towards new ways of functioning [49]. Moving away from lower-order, project-specific provider capabilities towards higher-order, generic eHealth capabilities might allow health care organizations to adapt to change, absorb new knowledge and innovate. Promoting patients’ eHealth literacy skills [37] now becomes a priority to enhance the continuity of mental health care.

### Growing awareness of eMental Health services

The majority of respondents stressed the need for national and local plans to combat perceptions of eMental Health as a “product” as opposed to a legitimate form of health service delivery. In order to be successful eMental Health care needs the clear endorsement and guidance from the health care system. Aligning interests across the multiple stakeholders remains a challenge but informants proposed strengthening implementation momentum by increasing the number of adjunctive and stepped-care uses of technology that do not replace the work of health care professionals so much as augment and extend it [50]. Informants also expressed a need for digital infrastructure to reduce duplication of efforts and make technologies more interoperable so providers can deliver more coordinated care. System-level change, it was suggested, is generally best accomplished by multi-level initiatives, delivered over a long period of time and modified in response to measured impact [51]. It was also recognized that improved awareness of available services would increase public demand and would be instrumental in the uptake and wider implementation of eMental Health care by government and professional practices alike.

### Limitations

Our small sample of informant interviews was an opinion collecting process rather than a consensus-building process. Interviewees did not have the opportunity to debate the issues with other decision-makers or other stakeholder groups. Notably, this study did not include the patient/public perspective but focused on individuals in leadership roles. However, this study serves as a basis for interactive dialogue around strategic priorities for future work. The heterogeneity of informant experiences, implementation efforts and health care settings adds to the robustness of our findings- particularly because of the international and cross-sectorial focus. However, the snowball sampling strategy may have biased the analyses in unpredictable ways.

The use of the Behaviour Change Wheel as opposed to other implementation frameworks (e.g., Reach Effectiveness Adoption Implementation Maintenance-[RE-AIM]) shaped the interpretation of results. We acknowledge that this model has certain underlying epistemological and ontological assumptions that shaped the way data was coded. However, the advantage of using a theory-driven and evidence-based model is that a priori specification of important constructs can increase interpretive power. In applying only the broadest level of the Behaviour Change Wheel (i.e., three core constructs of capacity, motivation, opportunity) we were able to incorporate emerging themes and simultaneously allow important new themes to emerge as well as explore disconfirming evidence [52].

Finally, it is important to note that results from informant interviews may not present the complex and contradictory nature of people’s views. People’s views are often context contingent, so opinions expressed in interviews vary according to how the interview is designed and presented to them [53]. However, by using a standard interview protocol and providing frequency and intensity analysis, we have attempted to be as transparent as possible in qualifying the pattern of results so the true diversity of opinions and views is evident.

## Conclusion

Large-scale, national initiatives designed to coordinate eHealth implementation are underway across the world and present important learning opportunities for where eMental Health fits within the larger eHealth movement. The significant commitments to eMental Health in some parts of the world suggest a growing community that recognizes nothing is going to happen just because of the enthusiasm of that community alone. We believe this novel study substantiates the need for decision-makers to lead sustained intersectoral efforts, to deliberate on these central implementation questions and to foster stronger global partnerships so that patient-centered solutions can be realized.

## Additional files

Additional file 1: Key Informant interview guide. (DOCX 21 kb)

Additional file 2: Key informants who provided consent to be listed on publications. (DOCX 46 kb)

## Abbreviations

BCW: Behaviour change wheel
EMR: Electronic medical record
ICT: Information communication technology
WHO: World Health Organization

## References

[1] WHO (2005). Fifty-eighth world health assembly: resolutions and decisions, annex.

[2] MHCC. e-Mental Health in Canada: Transforming the Mental Health System Using Technology, A Briefing Document. Ottawa: Mental Health Commission of Canada; 2014. http://www.mentalhealthcommission.ca/English/document/27081/e-mental-health-canada-transforming-mental-health-system-using-technology. Accessed 29 May 2017.

[3] APA (2010). American psychological association monitor.

[4] Steel Z, Marnane C, Iranpour C, Chey T, Jackson JW, Patel V, et al. The global prevalence of common mental disorders: a systematic review and meta-analysis 1980–2013. Int J Epidemiol. 2014;43(2):476–93. 10.1093/ije/dyu038 PMC3997379 24648481

[5] Huguet A, Rao S, McGrath PJ, Wozney L, Wheaton M, Conrod J (2016). A systematic review of cognitive behavioral therapy and behavioral activation apps for depression. PLoS ONE.

[6] Hollis C, Morriss R, Martin J, Amani S, Cotton R, Denis M (2015). Technological innovations in mental healthcare: harnessing the digital revolution. Br J Psychiatry.

[7] Gaebel W, Grobimlinghaus I, Kerst A, Cohen Y, Hinsche-Böckenholt A, Johnson B (2016). European Psychiatric Association (EPA) guidance on the quality of eMental health interventions in the treatment of psychotic disorders. Eur Arch Psychiatry Clin Neurosci.

[8] Naslund JA, Marsch LA, McHugo GJ, Bartels SJ. Emerging mHealth and eHealth interventions for serious mental illness: a review of the literature. J Mental Health. 2015;24(5):321–32. doi: 10.3109/09638237.2015.1019054. 10.3109/09638237.2015.1019054 PMC4924808 26017625

[9] Andrews G, Cuijpers P, Craske MG, McEvoy P, Titov N (2010). Computer therapy for the anxiety and depressive disorders is effective, acceptable and practical health care: a meta-analysis. PLoS ONE.

[10] Saddichha S, Al-Desouki M, Lamia A, Linden IA, Krausz M (2014). Online interventions for depression and anxiety - a systematic review. Health Psychol Behav Med.

[11] Arnberg FK, Linton SJ, Hultcrantz M, Heintz E, Jonsson U. Internet-delivered psychological treatments for mood and anxiety disorders: a systematic review of their efficacy, safety, and cost-effectiveness. PLoS ONE. 2014;9(5):e98118. 10.1371/journal.pone.0098118 PMC4028301 24844847

[12] Stephens-Reicher J, Metcalf A, Blanchard M, Mangan C, Burns J (2011). Reaching the hard-to-reach: how information communication technologies can reach young people at greater risk of mental health difficulties. Australas Psychiatry.

[13] Santana S, Lausen B, Bujnowska-Fedak M, Chronaki C, Kummervold PE, Rasmussen J, et al. Online communication between doctors and patients in Europe: status and perspectives. J Med Internet Res. 2010;12(2):e20. doi:10.2196/jmir.1281. 10.2196/jmir.1281 PMC2956231 20551011

[14] Wilson EV, Lankton NK (2004). Modeling patients’ acceptance of provider-delivered E-health. J Am Med Inform Assoc.

[15] WHO (2011). mHealth: new horizons for health through mobile technologies: second global survey on eHealth.

[16] Kessler RC, Angermeyer M, Anthony JC, De Graaf R, Demyttenaere K, Gasquet I (2007). Lifetime prevalence and age-of-onset distributions of mental disorders in the World Health Organization's World Mental Health Survey Initiative. World Psychiatry.

[17] Marcus MA, Westra HA, Eastwood JD, Barnes KL. What are young adults saying about mental health? An analysis of Internet blogs. J Med Internet Res. 2012;14(1):e17. doi: 10.2196/jmir.1868. 10.2196/jmir.1868 PMC3374526 22569642

[18] Eysenbach G (2001). What is e-health?. J Med Internet Res.

[19] Lewis C, Pearce J, Bisson JI (2012). Efficacy, cost-effectiveness and acceptability of self-help interventions for anxiety disorders: systematic review. Br J Psychiatry.

[20] Carr S, Lhussier M, Forster N, Geddes L, Deane K, Pennington M (2011). An evidence synthesis of qualitative and quantitative research on component intervention techniques, effectiveness, cost-effectiveness, equity and acceptability of different versions of health-related lifestyle advisor role in improving health. Health Technol Assess.

[21] Meurk C, Leung J, Hall W, Head BW, Whiteford H. Establishing and governing e-Mental health care in Australia: a systematic review of challenges and a call for policy-focussed research. J Med Internet Res. 2016;18(1):e10. doi: 10.2196/jmir.4827. 10.2196/jmir.4827 PMC4730106 26764181

[22] Batterham PJ, Sunderland M, Calear AL, Davey CG, Christensen H, Teesson M (2015). Developing a roadmap for the translation of e-mental health services for depression. Aust N Z J Psychiatry.

[23] Struthers A, Charette C, Bapuji SB, Winters S, Ye X, Metge C (2015). The acceptability of E-mental health services for children, adolescents, and young adults: a systematic search and review. Can J Commun Ment Health.

[24] Whitfield G, Williams C (2004). If the evidence is so good: Why doesn't anyone use them? A national survey of the use of computerized cognitive behaviour therapy. Behav Cogn Psychother.

[25] Ruwaard J, Kok RN (2015). Wild west eHealth: time to hold our horses?. Eur Health Psychol.

[26] Bates DW, Wright A. Evaluating eHealth: undertaking robust international cross-cultural eHealth research. PLoS Med. 2009;6(9):e1000105. 10.1371/journal.pmed.1000105 PMC2735779 19753106

[27] Li J, Talaei-Khoei A, Seale H, Ray P, Macintyre CR (2013). Health care provider adoption of eHealth: systematic literature review. J Med Internet Res.

[28] Mair FS, May C, O'Donnell C, Finch T, Sullivan F, Murray E (2012). Factors that promote or inhibit the implementation of e-health systems: an explanatory systematic review. Bull World Health Organ.

[29] Weiner BJ, Amick H, Lee SD (2008). Conceptualization and measurement of organizational readiness for change: a review of the literature in health services research and other fields. Med Care Res Rev.

[30] Lluch M (2011). Healthcare professionals’ organisational barriers to health information technologies—A literature review. Int J Med Inform.

[31] Lavis JN, Boyko JA, Oxman AD, Lewin S, Fretheim A. SUPPORT Tools for evidence-informed health Policymaking (STP) 14: organising and using policy dialogues to support evidence-informed policymaking. Health Res Policy Syst. 2009;7:1–8. doi: 10.1186/1478-4505-7-S1-S14. 10.1186/1478-4505-7-S1-S14 PMC3271825 20018104

[32] Whittaker R (2012). Issues in mHealth: findings from key informant interviews. J Med Internet Res.

[33] Jones RB, Ashurst EJ. Online anonymous discussion between service users and health professionals to ascertain stakeholder concerns in using e-health services in mental health. Health Informatics J. 2013;19(4):281–99 19. doi:10.1177/1460458212474908. 10.1177/1460458212474908 24255052

[34] Barak A, Grohol JM (2011). Current and future trends in Internet-supported mental health interventions. J Technol Hum Serv.

[35] WHO. Atlas of eHealth country profiles: the use of eHealth in support of universal health coverage: based on the findings of the third global survey on eHealth 2015. Geneva: World Health Organization; 2016. http://www.who.int/goe/publications/atlas_2015/en/. Accessed 29 May 2017.

[36] Baker S, Edwards R (2012). How many qualitative interviews is enough?.

[37] Guest G, Bunce A, Johnson L (2006). How many interviews are enough? An experiment with data saturation and variability. Field Methods.

[38] Strauss A, Corbin JM. Basics of qualitative research: grounded theory procedures and techniques. Newbury Park: Sage Publications, Inc.; 1990.

[39] Bradley EH, Curry LA, Devers KJ (2007). Qualitative data analysis for health services research: developing taxonomy, themes, and theory. BMC Health Serv Res.

[40] Michie S, van Stralen MM, West R (2011). The behaviour change wheel: a new method for characterising and designing behaviour change interventions. Implement Sci.

[41] Onwuegbuzie AJ. Effect sizes in qualitative research: a prolegomenon. Qual Quant. 2003;37(4):393–409.

[42] Sandelowski M, Barroso J, Voils CI (2007). Using qualitative metasummary to synthesize qualitative and quantitative descriptive findings. Res Nurs Health.

[43] Maxwell J (1992). Understanding and validity in qualitative research. Harv Educ Rev.

[44] Taylor SE, Crocker J, Fiske ST, Sprinzen M, Winkler JD (1979). The generalizability of salience effects. J Pers Soc Psychol.

[45] Wao HO, Dedrick RF, Ferron JM (2011). Quantitizing text: using theme frequency and theme intensity to describe factors influencing time-to-doctorate. Qual Quant.

[46] WHO (2016). From innovation to Implementation: eHealth in the WHO.

[47] Wells M, Mitchell KJ, Finkelhor D, Becker-Blease KA (2007). Online mental health treatment: concerns and considerations. Cyber Psychol Behav.

[48] Robert G, Greenhalgh T, MacFarlane F, Peacock R (2009). Organisational factors influencing technology adoption and assimilation in the NHS: a systematic literature review.

[49] Shaffer V, Rowsell-Jones A, Runyon B. The state of IT governance in healthcare delivery organizations and how to make it better; 2007.

[50] Castelnuovo G, Zoppis I, Santoro E, Ceccarini M, Pietrabissa G, Manzoni GM (2015). Managing chronic pathologies with a stepped mHealth-based approach in clinical psychology and medicine. Front Psychol.

[51] Canada Health Infoway (2013). A framework and toolkit for managing eHealth change: people and processes.

[52] Denzin N, Lincoln Y (2005). The SAGE handbook of qualitative research.

[53] Fischer P, Schulz-Hardt S, Frey D. Selective exposure and information quantity: how different information quantities moderate decision makers’ preference for consistent and inconsistent information. J Pers Soc Psychol. 2008;94(2):231–44. 10.1037/0022-3514.94.2.94.2.231 18211174

