# Supplementary material for: Implementation of eMental Health care: viewpoints from key informants from organizations and agencies with eHealth mandates
Source: BMC Med Inform Decis Mak. 2017 Jun 2;17:78. doi: 10.1186/s12911-017-0474-9 (PMC5455087; doi:10.1186/s12911-017-0474-9)
Supplement: Supplementary file 1 — Key Informant interview guide. (DOCX 21 kb) [file 12911_2017_474_MOESM1_ESM.docx]

**Additional File 1.** Key Informant interview guide.

***Part 1: Background***

1. How long have you been working in the field of eHealth? With [organization]?
2. What are your major responsibilities in your current position?
3. Can you describe your work and experience as it relates to eHealth?

***Part 2: Current eHealth Technologies***

1. How would you define eHealth?
2. Are you familiar with eHealth technologies to identify, treat and care for mental illnesses and disorders?

*If YES*

- 1. What is your current view of the credibility and quality of eHealth technologies for this patient population?
  2. Could you provide any examples of current eHealth technologies used to identify, treat and care for mental illnesses and disorders that you believe to be effective?
     1. How were these developed?
     2. How do these work?
     3. How do you know they’re effective?
     4. How are they evaluated?[56]

*If NO*

- 1. What eHealth technologies are you most familiar with?
  2. From your experience with these technologies, do you know of any important lessons that could be translated for identifying, treating, and caring for mental illnesses and disorders?
     1. Why or why not? [56]

1. Can you describe any eHealth innovation efforts being planned or currently underway in your organization/country?
   1. If yes, what are they and how would you define their overall effectiveness?
2. What would you do to improve eHealth measures currently in place?

***Part 3: Implementation Strategies***

1. In your country are you aware of an eHealth or eMental health strategy?
   1. If yes, can you broadly state your thoughts on them?
2. Is there a common national vision and/or policy concerning eHealth implementation in particular?
   1. If yes, what is it and do you feel your country follows these guidelines? What about your organization (if applicable)? Why or why not? [57]
3. What barriers do you think affect the implementation of eHealth innovations? What about facilitators?
4. What ways do you think current eHealth implementation strategies could be improved upon?

***Part 4: Adoption Readiness***

1. Do you personally use eHealth innovations in your practice/organization/personal life?
2. What obstacles exist in adapting to new eHealth strategies within health care systems/organizations?
   1. What could be done to overcome these? [58] What about facilitators?
3. What education/training would your staff/organizational members need in order to facilitate eHealth uptake? [58]
4. In what areas do you think eHealth technologies would have the largest benefit to health systems? Health care professionals?
5. What changes in governmental policies would be useful to support eHealth in your country/organization?
