# Supplementary material for: Implementation of eMental Health care: viewpoints from key informants from organizations and agencies with eHealth mandates
Source: BMC Med Inform Decis Mak. 2017 Jun 2;17:78. doi: 10.1186/s12911-017-0474-9 (PMC5455087; doi:10.1186/s12911-017-0474-9)
Supplement: Supplementary file 2 — Key informants who provided consent to be listed on publications. (DOCX 46 kb) [file 12911_2017_474_MOESM2_ESM.docx]

**Additional file 2.** Key informants who provided consent to be listed on publications.

Gavin Andrews, MD
Professor, University of New South Wales at St. Vincent’s Hospital
New South Wales, Australia

Victoria Betton, PhD
Programme Director, mHabitat, Leeds & York Partnership, NHS Foundation
Leeds, United Kingdom

Amy Bilderbeck, PhD
Post-doctoral researcher, Department of Psychiatry, University of Oxford, Warneford Hospital

Oxford, United Kingdom

Connie Coniglio
Provincial Executive Director, Children and Women’s Mental Health & Substance Use Programs, BC Mental Health & Substance Use Services (BCMHSUS)
British Columbia, Canada

Gareth Edwards
Director, Positive Thinking; Service User Academic, Centre for Mental Health Research, University of Auckland
Auckland, New Zealand

Nigel Fairley
Director, Area Mental Health Services, Capital & Coast District Health Board
Wellington, New Zealand

Theresa Fleming, PhD
Senior Lecturer, Department of Paediatrics & Psychological Medicine, Child and Youth Health, University of Auckland
Auckland, New Zealand

John Grohol, Psy.D
CEO & Founder, PsychCentral
Massachusetts, United States

David Gustafson, PhD
Founder, Center for Health Systems Research and Analysis (CHSRA)
Wisconsin, United States

Virginia MacEwan
Senior Project Manager, Mental Health Service Improvement, Ministry of Health
Auckland, New Zealand

Jennifer Martin, PhD
Programme Manager, NIHR MindTech, Institute of Mental Health, University of Nottingham Innovation Park
Nottingham, United Kingdom

Chris Moy, MB BS
General Practitioner, Australian Medical Association
South Australia, Australia

Hugh Norriss
Director of Strategy, Advocacy and Research, Mental Health Foundation of New Zealand
Auckland, New Zealand

Alex Ramsey, PhD
Research Associate, Centre for Technology and Behavioral Health
Missouri, United States

Clare Shann, PhD
Global Mental Health Lead
Movember Foundation
Victoria, Australia

Anil Thapliyal
CEO, HealthTRx Limited
Adjunct Professor in e-Mental Health, Centre for eHealth, Faculty of Health & Environmental Sciences, Auckland University of Technology
Auckland, New Zealand

Nickolai Titov, PhD
Associate Professor, Department of Psychology, Macquarie University
Co-Director, eCentreClinic
Director, Mindspot
New South Wales, Australia

Andrew Tugwell
Director, Health Promotion and Prevention, Provincial Health Services Authority
British Columbia, Canada

Christiaan Vis
Associate Researcher, Vrije Universiteit Amsterdam, Faculty of Behavioral and Movement Sciences
Amsterdam, Netherlands

Jennifer Zelmer
Executive Vice President, Clinical Adoption, Innovation and Consumer Health Initiatives, Canada Health Infoway
Ontario, Canada
